# The Genetics of Diabetic Nephropathy

**DOI:** 10.3390/genes4040596

**Published:** 2013-11-05

**Authors:** Eoin Brennan, Caitríona McEvoy, Denise Sadlier, Catherine Godson, Finian Martin

**Affiliations:** 1 Diabetes Complications Research Centre, Conway Institute of Biomolecular and Biomedical Research, School of Medicine and Medical Sciences, University College Dublin, Dublin, Ireland; E-Mails: caitriona.mcevoy@ucd.ie (C.E.); catherine.godson@ucd.ie (C.G.); 2 Mater Misericordiae Hospital, Dublin, Ireland; E-Mail: denise.sadlier@ucd.ie; 3 Conway Institute of Biomolecular and Biomedical Research, School of Biomolecular and Biomedical Sciences, University College Dublin, Dublin, Ireland; E-Mail: finian.martin@ucd.ie

**Keywords:** diabetes mellitus, diabetic nephropathy, genome-wide association study, single nucleotide polymorphism

## Abstract

Up to 40% of patients with type 1 and type 2 diabetes will develop diabetic nephropathy (DN), resulting in chronic kidney disease and potential organ failure. There is evidence for a heritable genetic susceptibility to DN, but despite intensive research efforts the causative genes remain elusive. Recently, genome-wide association studies have discovered several novel genetic variants associated with DN. The identification of such variants may potentially allow for early identification of at risk patients. Here we review the current understanding of the key molecular mechanisms and genetic architecture of DN, and discuss the merits of employing an integrative approach to incorporate datasets from multiple sources (genetics, transcriptomics, epigenetic, proteomic) in order to fully elucidate the genetic elements contributing to this serious complication of diabetes.

## 1. Background and Introduction

A large proportion of people with diabetes will develop microvascular complications. In addition to microvascular complications, the larger blood vessels become atherosclerotic and the risk for cardiovascular disease is 2–3 times higher compared to people without diabetes [1,2,3]. Diabetic nephropathy (DN) is the leading cause of end-stage renal disease, present in approximately 25%–40% of patients with long-standing diabetes, affecting kidney function and conferring additional risk of cardiovascular disease and mortality [4,5,6]. DN is a chronic disorder typically characterized by progressive albuminuria and a decline in renal function. Initial microalbuminuria typically develops to macroalbuminuria over the course of decades [7,8,9]. Crucially, DN is not clinically detectable until significant kidney damage has developed, highlighting the need to identify early-stage biomarkers. 

In contrast to diabetic retinopathy, which increases in incidence as diabetes progresses until almost all patients are affected [10], the incidence of DN peaks at 15–20 years after the onset of diabetes and declines thereafter, with a cumulative incidence of <30% after 25 years of diabetes [11,12]. Not all diabetics with microalbuminuria will progress to macroalbuminuria [13,14], and regression to normoalbuminuria has been reported in several studies [15,16]. Current therapies to treat DN include strict glycemic control and systemic blood pressure interventions targeting the renin-angiotensin system [17,18,19,20]. However, optimal blood pressure and glycemic control offer at best partial protection, and high risks of cardiovascular disease, end-stage renal disease and mortality persist [21].

**Figure 1:**
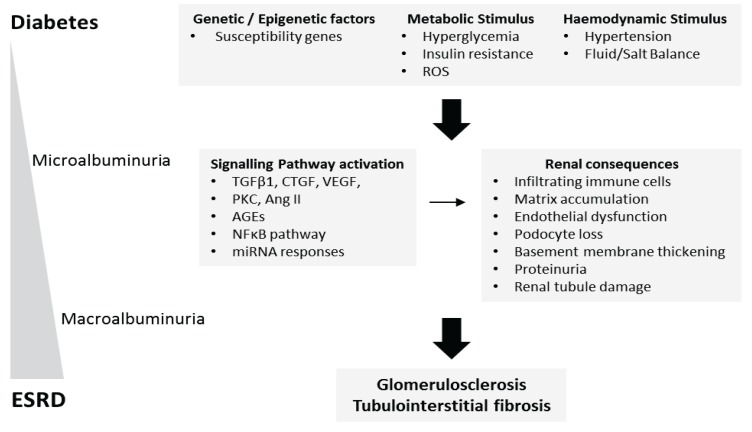
The pathophysiology of diabetic nephropathy. As a consequence of prolonged hyperglycaemia, diabetic nephropathy (DN) typically initiates as renal cellular hypertrophy and hyperfiltration, followed by progressive albuminuria and a decline in glomerular filtration rate (GFR). Microalbuminuria (urinary albumin excretion rate of 30–300 mg per day) develops 10–15 years after the onset of diabetes followed by macroalbuminuria (urinary albumin excretion rate of >300 mg per day) 15–25 years after diabetes onset. A combination of hyperglycaemia, inflammation, and hypertension drive the development and progression of DN. Presently, it is unclear why some diabetics are susceptible and others appear protected against the development of DN. Currently unknown causal and protective genetic variants have been suggested as one possible mechanism. At the cellular level, high glucose, Ang II, ROS, and profibrotic cytokines including TGF-β1, VEGF and CTGF have been identified as important modulators driving renal fibrosis. More recently, miRNA-mediated regulation and histone modifications have also been implicated in DN pathogenesis. Mutations in one or more key signaling pathways implicated in DN may act to suppress or drive DN progression.

During the development and progression of DN, chronic elevated blood glucose (hyperglycaemia) together with glomerular hypertension leads to renal inflammation, progressive glomerulosclerosis and tubulointerstitial fibrosis resulting in organ failure (Figure 1). High glucose, advanced glycation end-products (AGEs), Angiotensin II, and profibrotic growth factors including transforming growth factor-β (TGF-β) and connective tissue growth factor (CTGF) act on multiple cell types within the kidney to effect mesangial cell matrix expansion, podocyte cell loss, tubule epithelial cell apoptosis and dedifferentiation, macrophage recruitment and fibroblast activation [22,23,24,25,26,27,28]. Among the key modulators implicated in the pathogenesis of DN are oxidative stress, protein kinase C, activation of receptor of AGEs (RAGE) and the nuclear factor-κB (NF-κB) transcription factor family (Figure 1) [29,30,31,32]. As a result of the diverse functional roles of these resident renal cell populations, it is essential that the variations in gene expression patterns within these cell populations is completely understood in the context of the pathogenesis of DN. Furthermore, epigenetic mechanisms of gene regulation, including DNA methylation, histone modifications and micro-RNA (miRNA) are now being explored in DN [33]. 

There is evidence for a genetic susceptibility to nephropathy from case-control association and linkage studies, and more recently from genome-wide association (GWA) studies [34,35]. However, as is the case with the majority of complex diseases, identifying precise causal genetic variants contributing to DN has proven difficult. A better understanding of the heritable genetic factors underlying DN and its pathogenesis may lead to the discovery of new biomarkers of disease susceptibility and novel therapeutic strategies. For diseases with complex phenotypes such as DN, integration of multiple data sources promises an improved understanding of the disease pathophysiology. Here we review our present understanding of molecular drivers of DN and key *in vitro* and animal models of renal fibrosis, we discuss the underlying genetic subcomponent to DN, and finally we explore the merits of integrating data from multiple sources, including genetics, transcriptomic and epigenomic studies in order to define key targets and pathways worthy of investigation as potential therapeutic targets.

## 2. High Glucose in Diabetic Nephropathy

Hyperglycaemia is a major stimulus for the development of nephropathy in both type 1 and type 2 diabetes, and the most effective way to minimize the risk of diabetic complications is to maintain optimal glycemic control [36,37]. Several major mechanisms are believed to be responsible for the hyperglycaemia mediated pathological alterations observed in DN, including increased glucose flux through the hexosamine and polyol pathways, oxidative stress and overproduction of AGEs. As proposed by the Brownlee unifying hypothesis, there appears to be a pathogenic link between hyperglycaemia induced oxidative stress and other hyperglycaemia-dependent mechanisms of vascular damage [22]. Brownlee’s hypothesis proposes that mitochondrial superoxide production limits the flux of glucose through the entire glycolytic pathway, resulting in an increase in all upstream glycolytic intermediates. This increase in concentration of intermediates leads to increased activation of PKC isoforms, increased production of AGEs, and accelerated glucose flux through the polyol and hexosamine pathways.

Renal cells cultured in either physiologic glucose (5 mM) or glucose levels analogous to the diabetic environment (30 mM) are effective in mimicking cellular responses to hyperglycaemia. Studies investigating gene expression in primary human mesangial cells exposed to high glucose) have identified a large number of differentially regulated genes [38,39], including modulators of mesangial cell contractility, turnover and matrix metabolism, and genes reported previously to have increased expression in DN (e.g., fibronectin, thrombospondin, caldesmon). Several novel regulators have also been identified including IHG-1 and gremlin, both of which have been the subject of recent functional studies [40,41,42]. In recent years, miRNA responses in high glucose cultured animal and human renal cells has also been investigated [43,44]. Among those identified, miR-377 is induced by high glucose in both mouse and human mesangial cells, and drives increased fibronectin expression in these cells [45]. Alterations in epigenetic chromatin marks such as histone methylation, acetylation and ubiquitination have also been identified in kidney cells in response to the high glucose environment. Responsive epigenetic marks identified include H3Kme active and repressive marks [46] and H2A/H2B ubiquitination [47]. 

## 3. Profibrotic Growth Factors in Diabetic Nephropathy

The growth factor transforming growth factor-β1 (TGF-β1) is recognized as a central cytokine in the development and progression to DN. TGF-β1 is a member of the TGF-β superfamily of proteins, involved in a variety of cell processes including the control of cell growth, proliferation, differentiation and apoptosis [48,49]. TGF-β1 signal transduction is mediated via activation of canonical (SMAD) and non-canonical (p38MAPK, ERK, JNK, PI3K/Akt) pathways [49,50]. TGF-β is secreted by the majority of immune cells and acts as a potent immunoregulatory cytokine [48,50]. There is also evidence that elevated TGF-β levels can promote fibrosis in multiple organs, including the development of DN-associated glomerulosclerosis and tubulointerstitial fibrosis [51,52]. Interventions that reduce TGF-β1 activity have shown therapeutic benefit in renal fibrosis in type 1 and type 2 diabetes [53,54]. However, targeting TGF-β1 for therapeutic intervention is complicated by its dual role in inflammatory and immune processes. 

The concentration of TGF-β1 is highly variable and depends on the cellular microenvironment, and measurement of TGF-β1 concentrations within tissues is complicated by the fact that TGF-β1 is stored in the extracellular matrix as a latent form. While plasma and urine TGF-β1 levels have been measured in patients with diabetes and kidney disease, considerable differences are seen between studies [55,56]. 

TGF-β1 at concentrations of 1–10 ng/mL is routinely used to elicit appropriate fibrotic responses at the cellular level. RNA sequencing transcriptome analysis has identified approximately 2,000 genes differentially regulated in human renal tubule epithelial cells when challenged with high levels of TGF-β1 [57]. The role of tubule epithelial cells in kidney fibrosis is controversial with some studies suggesting kidney epithelia undergo epithelial-to-mesenchymal transition and migrate towards the interstitium in response to profibrotic cytokines including TGF-β1, thereby contributing towards the scar tissue forming myofibroblast population observed in kidney disease [58]. This theory has been challenged in recent years by lineage tracing studies suggesting that the contribution of renal epithelia to the myofibroblast population is minimal (5%), with resident interstitial and bone marrow-derived fibroblasts the main contributors to the myofibroblast pool [59]. TGF-β1 mRNA containing exosomes released by injured epithelial cells promote fibroblast activation [60], indicating that microparticle-mediated communication may be one possible mechanism through which renal epithelia contribute towards kidney fibrosis.

In addition to gene expression changes, there are several lines of evidence to suggest TGF-β1 induces aberrant alterations in epigenetic marks and miRNA expression. Histone acetylation has been suggested to have an important role in renal epithelial-mesenchymal transition, with treatment of renal epithelial cells with trichostatin A, an inhibitor of the histone deacetylase enzyme, shown to inhibit TGF-β1 induced epithelial-mesenchymal transition [61]. In addition, TGF-β1 treated mesangial cells increase levels of the histone methyltransferase SET7/9, which is associated with the expression of profibrotic genes in these cells [46]. TGF-β1 induces a loss of miR-192 expression in renal epithelial cells, resulting in increased expression of E-Box repressors ZEB1 and ZEB2, and silencing of E-cadherin expression [62,63,64]. More recently, TGF-β1 has been shown to suppress the let-7 miRNA family in renal epithelial cells resulting in upregulation of let-7 targets TGFβ receptor I, collagens, thrombospondin and the transcription factor HMGA2. Interestingly, treatment of cells with the pro-resolving lipid mediator lipoxin A4 reverses the loss of let-7c and attenuates the TGF-β1 profibrotic signal [65].

While TGF-β1 remains the most studied of the profibrotic cytokines considered as pathogenic mediators of DN, there are a multitude of additional profibrotic growth factors implicated in DN, including vascular endothelial growth factor (VEGF) [66] and connective tissue growth factor (CTGF) [67]. In human mesangial cells, both high glucose and TGF-β1 induce CTGF expression, mediated via TGF-β1-dependent and protein kinase C (PKC)-dependent pathways [67]. CTGF enhances expression of multiple extracellular matrix proteins observed in DN including collagens I and IV and fibronectin [39]. In an animal model of type 1 diabetes (STZ rat), CTGF renal expression is elevated at the time of mesangial expansion and proteinuria [39]. More recently, a study investigating the signaling interplay between CTGF and TGF-β1 in human mesangial cells identified upregulation of miR-302d and targeting of TGFβ receptor II in response to CTGF [68]. 

## 4. Animal Models of Diabetic Nephropathy

In 2001, the Animal Models of Diabetic Complications Consortium (AMDCC) was set up by the National Institutes of Health to develop murine models of diabetic micro and macrovascular complications that completely replicate the human diseases [69]. Currently, there is a lack of reliable animal models that mimic nephropathy in human type 1 or type 2 diabetes, and the debate as to what exactly is the best animal model is somewhat controversial. Chemical agents such as streptozotocin (STZ) can selectively damage the insulin-producing beta-cells in the pancreas resulting in hyperglycaemia and are long recognized as important tools for developing animal models of diabetic complications [70,71,72]. However while this model induces kidney hypertrophy and mesangial expansion, it does not progress to more advanced renal disease seen in humans which is characterized by a loss of glomerular filtration, overt proteinuria, advanced structural lesions and tubulointerstitial fibrosis [70,71]. Mouse models of endothelial dysfunction, with a targeted mutation in the Nos3 gene encoding endothelial nitric oxide synthase are recognized as one of the more robust models of advanced renal disease in diabetes; these animals exhibit a decline in glomerular filtration and tubulointerstitial fibrosis [71]. Recently developed mouse models with a leptin receptor mutation develop type 2 diabetes, hypertension, obesity and on the db/db or BTBR ob/ob background develop proteinuria, reduced glomerular filtration, mesangial matrix expansion and podocyte loss [73], thereby recapitulating nephropathy in type 2 diabetes. Finally, the unilateral ureteral obstruction (UUO) rat model, generated through ligation of the ureter, results in marked renal hemodynamic changes, followed by tubular injury, cell death and interstitial macrophage infiltration, and is considered an important model for tubulointerstitial fibrosis [74,75,76,77]. 

While no single-animal model exists that recapitulates all of the pathological features of established human nephropathy in type 1 and type 2 diabetes, these animal models have provided valuable information regarding many aspects of DN including pathophysiology and implicated genes. Microarray analysis of glomerular gene expression profiles in diabetic rats has identified numerous signaling networks implicated in human DN, including extracellular matrix interacting genes, renin-angiotensin, TGF-β1, Smad and NF-κB pathways [78,79,80,81]. 

## 5. Human Studies in Diabetic Nephropathy

### 5.1. Gene Expression Studies in DN

Genome-wide transcriptome analysis of human tissues using expression arrays or next-generation sequencing is a common strategy used to gain insight into disease pathogenesis and identification of biomarkers. Comprehensive studies analyzing gene expression in renal biopsies from DN patients are somewhat limited due to the invasive nature and risks associated with biopsy. In recent years, several gene expression studies have been performed on DN patient renal biopsies, leading to the identification of potential causal pathways. The first such study performed by the European Renal cDNA Bank focused on transcriptome analysis of tubulointerstitial tissue from DN patients of Caucasian backgrounds. It identified components of the NF-κB regulated transcriptome to be upregulated in DN [32]. More recently, a comprehensive microarray study of DN kidney biopsies from ethnically diverse patients microdissected into glomerular and tubulointerstitial compartments identified both common and distinct expression networks and pathways implicated in disease, including the complement cascade, RhoA, Cdc42, integrin, integrin-linked kinase, and vascular endothelial growth factor signaling in DN glomeruli [82].

An important consideration when using *in vitro* and *in vivo* model systems is how accurate these models are in mimicking human DN. An example of where *in vitro* models can inform human disease is seen in a recent study of human kidney epithelial cells exposed to albumin identifying 231 differentially expressed genes, which were subsequently used as a classifier set to distinguish control *versus* IgA nephropathy biopsies [83]. A similar strategy was utilized to correlate whole-transcriptome expression data from TGF-β1 stimulated kidney epithelial cells with expression data from renal biopsies of patients with DN *versus* healthy donor controls. This study identified a cohort of 179 co-regulated transcripts [57]. Pathway analysis of the genes regulated by TGF-β1 in this model system identified an enrichment of NF-κB pathway genes, which closely mimics what is observed in human DN biopsies.

### 5.2. Epigenetic Studies in DN

Emerging evidence suggests that epigenetic mechanisms play a role in the development of renal diseases such as DN. Clinical and experimental studies demonstrate the occurrence of a hyperglycemic memory due to previous exposure to high glucose levels, resulting in persistently increased risk for DN [84]. Proposed epigenetic mechanisms include glucose-induced effects on histone modifications leading to modulation of vascular gene expression, which persists despite a return to normoglycemia [85]. Monocytes from diabetic patients also exhibit histone modifications including changes H3K9me2 and H3K4me2, which are associated with immune and inflammatory pathways [86]. Alterations in DNA methylation profiles has also been reported, with a genome-wide DNA methylation analysis of type 1 diabetes patients with or without nephropathy identifying CpG sites where the degree of methylation correlated with time to development of DN [87]. Due to the cell-specific nature of epigenetic marks several issues arise when using patient samples and cell model systems. Firstly, while the identification of predictive epigenetic marks in DN patient blood offer excellent high-throughput and non-invasive screening potential, the functional relevance of these marks to the distant kidney, composed of many different cell types each with potentially distinct epigenetic profiles, is unclear. Secondly, the validity of cell lines in DNA methylation studies in DN has been called into question. A comparison of DNA methylation profiles in peripheral blood leukocytes *versus* lymphoblastoid cell lines generated from the same DN patients identified differential methylation in 8% of the promoter CpG islands assessed, raising doubts over the utility of EBV-transformed cell lines [88]. Finally, the utility of short-term stimulations routinely used in *in vitro* models of DN to investigate epigenetic marks is also questionable. For example, while there is abundant evidence of global gene expression changes in kidney cells cultured in the presence of profibrotic growth factors within 24 h, there is also evidence that DNA methylation changes may not become apparent at these genes for several days [89]. As a result, extended time-course experiments will be essential when investigating epigenetic marks in cell models of DN.

miRNA profiling in a number of diseases, including diabetic complications has also been performed. Considering the cell-specific nature of miRNA expression, the vast majority of studies implicating miRNA signaling cascades in the pathogenesis of DN have focused on individual resident renal cell populations (epithelial, mesangial, podocyte) using the *in vitro* and animal model systems described in this review. Among the numerous miRs implicated in diabetic kidney disease are miR-192, miR-200, let-7c, miR-21, miR-29 and miR-37 [45,64,90,91,92]. miR-192 is perhaps the most studied micro-RNA in human kidney disease, and the precise role of this miR remains unclear due to conflicting reports. While some studies have reported increased renal miR-192 expression in diabetic mice and also in mouse and rat kidney cells in response to TGF-β1 [63,93], other studies have reported the opposite trend [64]. The observed differences are likely to be due to different models being used, cell-specific responses, and cell culture conditions. Analysis of miRNA expression levels in renal biopsies reported a loss of miR-192 which correlated with severity of kidney disease in DN patients [43], and several studies have also reported loss of miR-192 in human renal epithelial cells stimulated with TGF-β1 [43,65]. Despite the difficulties associated with interpreting these results, it is clear that miR-192 likely plays an important role in kidney disease. More recently, several studies have explored the potential use of urinary miRNA expression levels in DN patients as biomarkers of disease progression [94,95]. Presently, there are several limitations to miRNA therapeutics in DN and other human diseases including the fact that miRNAs may target hundreds of transcripts in addition to a desired target transcript, and precise delivery of these miRNAs to specific sites of kidney injury will be required. Interestingly, a miR-34 mimic which targets known oncogenes has recently entered a phase 1 trial in patients with liver cancer [96].

## 6. Genetic Architecture of Diabetic Nephropathy

### Gene Discovery in Diabetic Nephropathy

Early evidence for heritability of DN came from family-based studies reporting clustering of nephropathy in sibling pairs with type 1 and type 2 diabetes [97,98,99]. Likewise, a parental history of hypertension or cardiovascular disease is predictive of DN [100,101]. Despite the fact that linkage studies have identified several chromosomal regions linked with nephropathy in type 1 and type 2 diabetes [102,103,104,105,106,107,108,109,110], no linkage peaks have been robustly reproduced across multiple studies. Candidate genes investigated for association studies in DN have primarily been selected based on results from these genome-wide linkage scans and putative biological role in pathways implicated in the pathogenesis of DN. Thus, candidate genes investigated for association with DN include: profibrotic growth factors (CTGF, TGFβ1, TGF-β receptors 2 and 3, VEGF) [111,112,113,114,115]; BMPs and antagonists (BMP-2,4,7, gremlin) [40,116]; inflammatory molecules (ICAM1, MCP1, RAGE) [117,118,119]; and dyslipidemia-related genes [120]. 

Angiotensin-converting enzyme (ACE) inhibitors are routinely used antihypertensive agents in the treatment of DN, and the ACE insertion/deletion (I/D) polymorphism is perhaps the most well-studied candidate gene in DN [121,122,123,124,125,126]. Despite conflicting results reported for this polymorphism, a meta-analysis of 47 studies showed that the ‘II genotype’ has a potentially protective effect. It confers a markedly lower risk of developing DN than seen in carriers of the “D allele” [127]. SNPs within the angiotensinogen (AGT) and angiotensin II receptor, type 1 (AT1) genes are also associated with a higher risk of developing nephropathy [128]. However, as is the case with many complex disease genetic studies, where candidate gene associations have been reported in DN, consistent replication across multiple studies has been limited, suggesting that large-scale genome-wide efforts will be required to identify putative candidate genes. 

Genome-wide association (GWA) studies have identified more than 100 genetic variants associated with type 1 and type 2 diabetes [129,130,131,132,133,134], with successful GWA studies also reported for non-diabetic kidney diseases such as IgA, ANCA-associated and idiopathic membranous nephropathy [135,136,137,138,139]. However, GWA studies in DN have so far proven less fruitful, with robust replication of many initial associations not forthcoming. Reasons for a lack of corroboration include a combination of effects due to small sample sizes, modest gene effect sizes, population stratification and lack of consistency with respect to patient recruitment criteria, phenotype definition and statistical analysis. It is now apparent that in order to fully elucidate the genetic elements contributing to nephropathy in diabetes, collaborative efforts merging sample collections for analysis in GWA studies are required.

In 2009, the U.S. Genetics of Kidneys in Diabetes (GoKinD) GWA study analyzed SNPs in 820 DN case subjects and 885 control subjects with type 1 diabetes but no evidence of DN [34]. Although no risk variant achieved genome-wide significance, the primary association analysis identified 11 SNPs representing four distinct chromosomal regions. Among the associated variants was a SNP located in the promoter of the FRMD3 gene. FRMD3 (FERM domain containing 3) belongs to the family of band 4.1 proteins, encoding adaptors of plasma membrane receptors to the actin cytoskeleton. Multiple SNPs in the FRMD3 gene have been reported to be associated with nephropathy in both type 1 and type 2 diabetes in European, African and Asian populations [140]. Interestingly, a recent study has defined a putative functional role for the promoter SNP rs1888747 [140,141]. Here, the FRMD3 transcript was shown to be co-expressed with BMP signaling pathway components in renal biopsies of DN patients. In the same study, bioinformatic analysis of the FRMD3 and BMP family promoter sequences identified shared transcription factor binding sites, suggesting co-regulation with the BMP family. A comprehensive meta-analysis for genetic variants associated with DN, selecting only variants where associations were independently reproduced, identified 24 loci of interest including ACE and FRMD3 polymorphisms [142]. In 2012 the Genetic of Nephropathy—an International Effort (GENIE) consortium completed a large meta-analysis and GWA study, consisting of 6,366 participants of European ancestry with type 1 diabetes, with and without nephropathy [35]. Associated variants were identified in several novel candidate genes: AFF3, ERBB4, 15q26, and functional data implicated AFF3 in TGF-β1 mediated renal tubule fibrosis. Variants in AFF3 have been reported in a GWA study for type 1 diabetes [129,143], and ERBB4 has also been shown to have a potentially important biological role in kidney tubule integrity [144,145]. More recently, this consortium has also identified a sex-specific genetic variant on chromosome 2 strongly associated with end-stage renal disease in female patients with type 1 diabetes [146]. While independent replication of this signal will be required, this robustly associated genetic variant may explain how some diabetic women lose their protection against end-stage renal disease.

Despite the promising replication observed in a previous meta-analysis [142], this consortium was unable to replicate most of the previously reported genetic associations for nephropathy in type 1 diabetes [147]. Importantly, additional consortia have now been established to perform GWA studies in DN, including SUMMIT [148]. Future directions to identify genetic variants associated with diabetic kidney disease will require replication of GWAS signals to determine whether these are indeed ‘true’ DN-associated variants, merging of these datasets to increase statistical power, investigation of rare low-frequency variants not captured by standard GWA study approaches (exome chips), and use of extreme phenotype cohorts in association studies.

## 7. Integrating Multi-Layered Datasets in Diabetic Nephropathy

While association and linkage studies provide an enormous amount of information, incorporation of multiple sources of evidence is necessary to decipher biological processes linked to DN. It is through integration of multiple layers of evidence (genome-wide association studies, transcriptomics, epigenomics, proteomics and metabolomics) that promising candidate genes and pathways will be discovered. There are now several web resources available which collate published renal disease datasets. Nephromine [149], a resource of renal gene expression profiles provides current information on existing datasets related to DN and other kidney disease phenotypes. Others include: the Kidney and Urine Pathway Knowledge Base [150,151] which contains experimental data (mRNA, proteins and miRNA) coming from published articles related to kidney physiology and pathology; the Human Kidney and Urine Proteome Project, an initiative project of the Human Proteome Organization to analyze human kidney and urine by proteomics [152]; and EuReGene, the European Renal Genome Project [153], an atlas of gene expression in the developing and adult mouse kidney.

**Figure 2:**
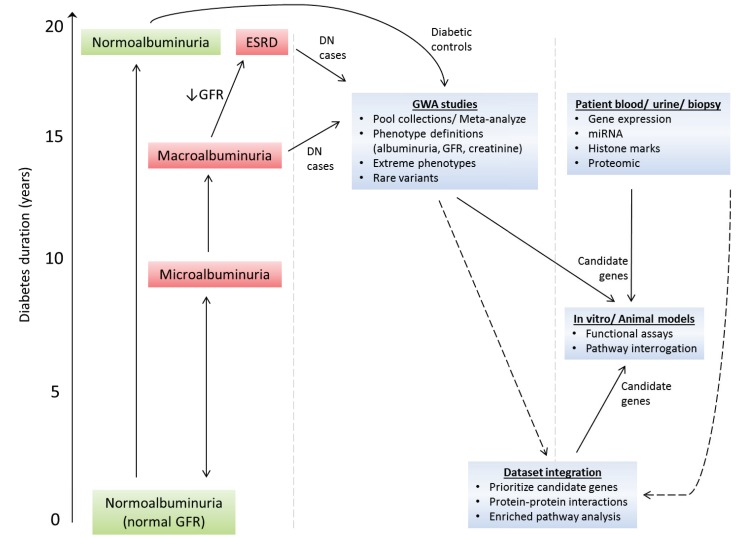
Unravelling the genetic and molecular mechanisms that cause DN development and progression. The clinical course of DN is relatively well defined. Diagnosis of DN is complicated by the fact that: (**a**) patients with microalbuminuria may undergo spontaneous regression to normoalbuminuria; and (**b**) glomerular filtration rate (GFR) and albuminuria may progress independently of each other, *i.e.*, patients may have micro- or macro-albuminuria even though their GFR is normal. Macroalbuminuria is usually associated with reduced GFR. Large scale genome-wide association (GWA) studies in DN are now feasible—future direction will require merging of patient cohorts, use of alternative phenotype definitions to the standard albumin excretion rate definition (e.g., estimated GFR, serum creatinine), analysis of extreme phenotype cohorts, and rare-variant GWA studies. To prioritize candidate genes from GWA studies in DN, these datasets will be integrated with whole-genome expression, proteomic, micro-RNA and epigenomic markers datasets using newly developed integrative tools to merge such datasets. As prioritized candidate genes are identified, functional analysis of these genes will be performed in established cell and animal models of DN.

A recent study merged publicly available omics data specific for DN along with DN-associated data retrieved from scientific literature, and subsequently mapped these data onto a protein interaction network [154]. These data identified the renin-angiotensin, complement and coagulation cascade, and PPAR pathways as primary targets worthy of further investigation in DN. In recent years, several web-applicable tools have been developed to facilitate the merging of multiple omics datasets to identify candidate genes, including ENDEAVOUR and CANDID [155,156]. More recently, MetaRanker has been developed to identify susceptibility genes based on meta-analysis of heterogeneous data from GWA studies, protein-protein interaction screens, disease similarity, linkage studies, and gene expression experiments which is used to prioritize candidate genes [157,158]. Approaches such as these that incorporate multiple layers of evidence will most likely identify highly relevant DN-associated genes (Figure 2).

## 8. Discussion and Conclusions

Diabetes and associated complications represent a significant health and economic burden, and given the emerging epidemics of obesity and diabetes in children and adolescents these increases in prevalence are expected to continue. Diagnosis of DN is typically made using clinical criteria (albuminuria, serum creatinine) rather than invasive renal biopsy. However, DN is not clinically detectable until significant kidney damage has developed, highlighting the need to identify early-stage biomarkers. 

Despite the large body of evidence for a heritable genetic component to nephropathy in type 1 and type 2 diabetes, the underlying genetic mechanisms remain poorly understood, with no robust DN candidate genes yet identified. While the majority of candidate gene studies in DN have tended to focus on strong biological candidates, it is interesting to note that as data emerges from GWA studies the majority of signals reside in novel genes, often with little known on their precise biological role. This would suggest that novel risk factors and pathways may play an important role in the progression and development of DN. It is likely that mutations in multiple genes distributed across the genome, each with a modest effect size, may be causal or protective factors in the development and progression of DN. As is the case with many complex diseases, robust replication of DN-associated gene variants across multiple populations has been limited. Therefore, an essential step towards identifying variants associated with nephropathy in diabetes will involve: merging patient cohorts across multiple populations to increase sample size to detect gene associations of modest effect; investigating rare variants which are not captured by current GWA studies; and defining clear disease phenotype criteria.

It is now clear that there is a need for improved or alternative phenotype definitions to the standard trait for genetic studies of DN—albuminuria. DN disease progression is variable; some patients progress relatively fast, whereas others do not, which could indicate a potentially heterogeneous disease. We now know that proteinuria is more variable in DN than initially thought, with regression from microalbuminuria to normoalbuminuria frequently observed during the early stages of DN [14], and studies reporting advanced diabetic kidney disease in the absence of proteinuria [159]. More recently, renal decline as measured by loss of eGFR has been reported in type 1 diabetics with normoalbuminuria, suggesting that this early renal decline precedes and drives progression towards microalbuminuria and macroalbuminuria [160]. As seen in a recently completed large-scale GWA study in type 1 DN, no single locus reached genome-wide significance when the accepted phenotype definitions were used to define DN cases (macroalbuminuria or end-stage renal disease) and diabetic controls with normal albumin excretion [35]. In the same study, when the DN case definition was modified to include only diabetic end-stage renal disease patients, *i.e.*, the most extreme cases, genome-wide significant signals were identified. Independent replication and fine-mapping of these loci will be essential to determine whether these are indeed ‘true’ end-stage renal disease signals. As a result, future GWAS in DN will consider additional phenotype definitions focusing on quantitative traits including serum creatinine and glomerular filtration rate.

Due to significant technological advances in recent years the cost of performing whole-transcriptome profiling has become more feasible, allowing for further investigation into the mechanisms driving this serious complication of diabetes. Presently, a large body of research in DN is focused on *in vitro* and animal models of DN. However, the lack of animal models that mimic human DN is a major obstacle to be overcome. While these model systems have proven to be invaluable tools in aiding our understanding of the pathophysiology of DN, there is a need for a more comprehensive analysis of human material (biopsy, urine, blood) from DN patients. Transcriptomic profiling via RNA sequencing, epigenomic profiling, and proteomic analysis of primary resident cell types and kidney compartments will be required to comprehensively map responsive markers during the development and progression of DN. As the wealth of data generated from these high-throughput techniques are integrated with GWA study data, and incorporated with additional layers of evidence, emerging computational tools will be utilized to define enriched gene pathways and networks.
